# *In Vivo* Translatome Profiling in Spinal Muscular Atrophy Reveals a Role for SMN Protein in Ribosome Biology

**DOI:** 10.1016/j.celrep.2017.10.010

**Published:** 2017-10-24

**Authors:** Paola Bernabò, Toma Tebaldi, Ewout J.N. Groen, Fiona M. Lane, Elena Perenthaler, Francesca Mattedi, Helen J. Newbery, Haiyan Zhou, Paola Zuccotti, Valentina Potrich, Hannah K. Shorrock, Francesco Muntoni, Alessandro Quattrone, Thomas H. Gillingwater, Gabriella Viero

**Affiliations:** 1 Institute of Biophysics, CNR Unit at Trento, Via Sommarive 18, 38123 Povo (Trento), Italy; 2 Centre for Integrative Biology, University of Trento, Via Sommarive 9, 38123 Povo (Trento), Italy; 3 Euan MacDonald Centre for Motor Neurone Disease Research, University of Edinburgh, Hugh Robson Building, 15 George Square, EH8 9XD Edinburgh, UK; 4 Edinburgh Medical School: Biomedical Sciences, University of Edinburgh, Hugh Robson Building, 15 George Square, EH8 9XD Edinburgh, UK; 5 Dubowitz Neuromuscular Centre, Great Ormond Street Institute of Child Health, University College London 30, Guilford Street, WC1N 1EH London, UK

**Keywords:** neurodegeneration, motor neuron disease, ribosome, translatome, polysomal profiling, spinal muscular atrophy, SMN

## Abstract

Genetic alterations impacting ubiquitously expressed proteins involved in RNA metabolism often result in neurodegenerative conditions, with increasing evidence suggesting that translation defects can contribute to disease. Spinal muscular atrophy (SMA) is a neuromuscular disease caused by low levels of SMN protein, whose role in pathogenesis remains unclear. Here, we identified *in vivo* and *in vitro* translation defects that are cell autonomous and SMN dependent. By determining in parallel the *in vivo* transcriptome and translatome in SMA mice, we observed a robust decrease in translation efficiency arising during early stages of disease. We provide a catalogue of RNAs with altered translation efficiency, identifying ribosome biology and translation as central processes affected by SMN depletion. This was further supported by a decrease in the number of ribosomes in SMA motor neurons *in vivo*. Overall, our findings suggest ribosome biology as an important, yet largely overlooked, factor in motor neuron degeneration.

## Highlights

• Polysomal profiling reveals translation defects in SMA mice
• Translation defects are SMN dependent and cell autonomous
• Translation efficiency alterations highlight defects in ribosome biology
• The number of axonal ribosomes is decreased in SMA *in vivo*

## Graphical Abstract

**Figure:**
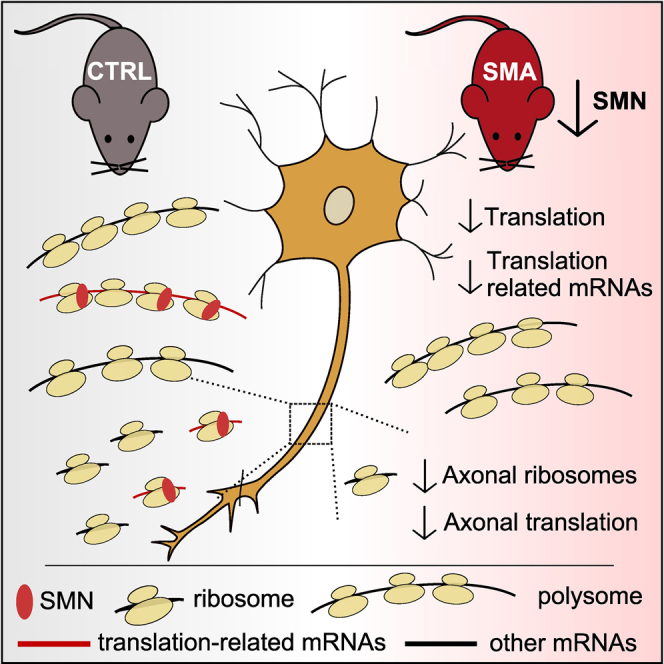

## Teaser

Bernabò et al. analyzed translation in a mouse model of spinal muscular atrophy and identified translation defects that arise early in pathogenesis, are cell autonomous, and are accompanied by a low number of axonal ribosomes in diseased nerves. Their findings highlight ribosome biology as a central hallmark of the disease.

## Introduction

Neurons are highly specialized cells, reliant on precise spatial and temporal control of translation to maintain their unique anatomy and physiology. Local control of protein synthesis allows neurons to regulate axonal outgrowth and growth cone dynamics during development and to control cellular processes required for maintaining neuronal homeostasis throughout the lifespan (Jung et al., 2014). It is therefore not surprising that dysregulation of translation has been linked to the pathogenesis of several neurological diseases. For example, genetic studies of familial and sporadic forms of motor neuron diseases have identified mutations in genes involved in RNA biology as active contributors to disease pathogenesis (Brinegar and Cooper, 2016, Cookson, 2016, Renton et al., 2014, Sephton and Yu, 2015). It remains unclear, however, why defects in ubiquitously expressed components of RNA metabolism lead to defects that are largely restricted to selected cell populations, and the extent to which changes in translation play a causative role in neurodegeneration *in vivo* remains to be fully determined.

Spinal muscular atrophy (SMA) is the most common genetic cause of infant mortality (Lefebvre et al., 1995, Lunn and Wang, 2008), where a loss of lower motor neurons leads to atrophy of skeletal muscle. In the most common and severe cases (type I), onset occurs ∼6 months of age (Lunn and Wang, 2008). SMA is caused by low levels of full-length survival of motor neuron protein (SMN). Humans have two copies of the gene encoding SMN: *SMN1* and *SMN2*. 95% of SMA cases are caused by homozygous deletion of *SMN1*. *SMN1* and *SMN2* are 99.9% identical, with just one base pair difference (Lorson et al., 1999), resulting in skipping of exon 7 in ∼85%–90% of *SMN2* transcripts. Without exon 7, the *SMN2*-derived protein is quickly degraded, leaving only low levels of functional SMN protein (Burghes and Beattie, 2009, Tisdale and Pellizzoni, 2015).

Several lines of evidence suggest that SMN protein is involved in the development and maintenance of motor neuron growth cones, axon, and neuromuscular junctions (Farrar et al., 2017) mediated, at least in part, through its role in regulating ubiquitin homeostasis (Groen and Gillingwater, 2015, Wishart et al., 2014). In concert with other RNA binding proteins, SMN forms complexes in the nucleus and cytoplasm of neurons (Donlin-Asp et al., 2016, Fallini et al., 2012). As such, SMN protein has housekeeping roles in the assembly of ribonucleoprotein (RNP) complexes, including the U3 snoRNA (Wehner et al., 2002) required for 18S ribosomal RNA biogenesis (Dragon et al., 2002) and the small nuclear RNP (snRNP) required for processing of histone pre-mRNAs (Tisdale et al., 2013). In addition, SMN localizes to dendrites, synapses, and axons *in vivo* and *in vitro* (Béchade et al., 1999, Jablonka et al., 2001, Wishart et al., 2014), where it is part of messenger RNP (mRNP) complexes with roles in mRNA transport (So et al., 2016, Zhang et al., 2008). These cellular processes are tightly linked to local translation, suggesting that SMN may play a pivotal role in its regulation. Indeed, SMN has been found to associate with polyribosomes *in vitro* (Sanchez et al., 2013) and in the rat spinal cord (Béchade et al., 1999). Moreover, components of the translation machinery are mislocalized in SMN-depleted cells (Gabanella et al., 2016), and localization and translation of specific mRNAs are altered in primary neurons derived from SMA mice (Fallini et al., 2011, Fallini et al., 2016, Rossoll et al., 2003). However, a direct link between SMN and the regulation of translation *in vivo* has not yet been demonstrated.

Here, we used polysomal profiling to investigate the role of SMN in translation in an established mouse model of SMA. We show that SMN depletion leads to widespread perturbations in translation, thereby identifying a key role for SMN in translation and ribosome biology *in vivo*.

## Results

### Polysomal Profiling Reveals Translation Defects in SMA

**Figure 1:**
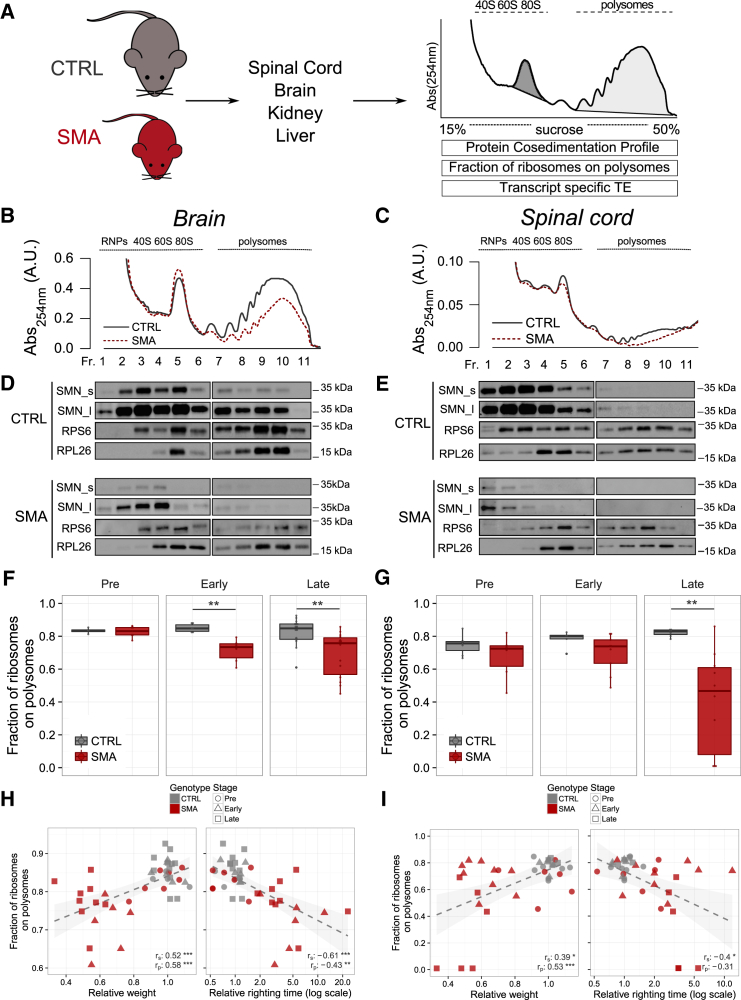
Translation Is Impaired in Symptomatic SMA Nervous Tissues (A) Experimental design and analyses using polysomal profiles from control (CTRL) and SMA mouse tissues. (B and C) Sucrose gradient absorbance profiles from CTRL and SMA brains and spinal cords (late symptomatic). (D and E) Co-sedimentation profiles of SMN and ribosome markers RPS6 and RPL26 under the corresponding sucrose gradient. The signal of SMN along the profile is shown for short (SMN_s) and long (SMN_l) exposure times of acquisition. (F and G) Comparison between the fraction of ribosomes in polysomes (FRP) in CTRL and SMA mouse brains (F) and spinal cords (G) at three stages of disease (brain: pre-symptomatic, CTLR n = 4, SMA n = 7; early symptomatic: CTLR n = 6, SMA n = 6; late symptomatic: CTRL n = 16, SMA n = 14; spinal cord: pre-symptomatic: CTRL n = 7, SMA n = 6; early symptomatic: CTLR n = 5, SMA n = 9; late symptomatic: CTLR n = 7, SMA n = 10, ^∗^p < 0.05, ^∗∗^p < 0.01, ^∗∗∗^p < 0.001, two-tailed t test). (H and I) Relationship between body weight (left) or righting time (right) and the corresponding FRP, obtained from CTRL and SMA mouse brains (H) and spinal cords (I). Each point corresponds to one mouse. Spearman and Pearson correlations between are indicated (^∗^p < 0.05, ^∗∗^p < 0.01, ^∗∗∗^p < 0.001, correlation test). See also Figure S1 and Data S1.

To test the hypothesis that low levels of SMN lead to defects in translation *in vivo*, we performed polysomal profiling on brain, spinal cord, kidney, and liver harvested from the “Taiwanese” SMA mice (Hsieh-Li et al., 2000, Wishart et al., 2014) at pre-, early-, and late-symptomatic stages of disease. Since one major challenge in studying neurodegenerative diseases such as SMA is to distinguish true pathogenic changes—occurring early in pathogenesis—from downstream cellular phenotypes that represent later-stage consequences of the disease (Bäumer et al., 2009), we did not include disease end-stage mice, thereby avoiding secondary, non-specific changes. We performed polysomal profiling to gather information about the translation state in SMA, both *in vitro* and *in vivo*. We determined the following under both physiological and diseased conditions: (1) the fraction of ribosomes engaged on polysomes, revealing the overall level and efficiency of translation (Brina et al., 2015), (2) the protein co-sedimentation profile with ribosomes and polysomes (Darnell et al., 2011, Tiedje et al., 2012), and (3) the transcriptome-wide analysis of RNAs associated with polysomes (i.e. the translatome, POL-seq) by next-generation sequencing (NGS) (King and Gerber, 2016) (Figure 1A). Graphical representations of typical polysome profiles are shown in Figure 1B for brain and in Figure 1C for spinal cord. The first peak contains free cytosolic light components (RNPs), and the subsequent peaks include ribosomal subunits (40S and 60S) and monosomes (80S), all associated with non-translating particles. The remaining peaks of the profile represent polysomes, which sediment with high sucrose concentrations and contain the RNAs associated with ribosomes.

The cytoplasmic localization of SMN, alongside its known role in mRNA transport along the axon and its potential association with both ribosomal proteins (Fuller et al., 2010) and polysomes (Béchade et al., 1999, Sanchez et al., 2013), prompted us to confirm its association with polysomes *in vivo*, determining the co-sedimentation profiles of SMN in control and SMA brain and spinal cord by immunoblotting (Figures 1D and 1E, respectively). In control mice, we observed co-sedimentation of SMN with the 40S ribosomal subunit, 80S monosomes, and polysomes in brain (Figure 1D) and spinal cord (Figure 1E). To further study the association of SMN with ribosomes/polysomes, we determined the co-sedimentation profiles of SMN in the presence of EDTA, which dissociates ribosomes into small and large subunits, finding that SMN moved toward lighter fractions (Figure S1A). Moreover, when a sub-cellular fractionation coupled to high-salt wash was performed, SMN was found to be tightly associated to the ribosome fraction (Figure S1B). In SMA mouse tissue, in addition to an overall reduction in SMN levels, co-sedimentation analysis demonstrated a corresponding decrease of SMN co-sedimentation with both monosomes and polysomes (Figures 1D and 1E). Moreover, in SMA spinal cord, SMN moved toward lighter fractions that correspond with ribonucleoparticles (Figure 1E). Thus, cytoplasmic SMN partially co-sediments with translation machinery *in vivo*, while in SMA, SMN is depleted from ribosomal fractions and moves to non-ribosome-associated fractions.

When comparing polysomal profiles from late-symptomatic SMA and age-matched control tissue (Figures 1B and 1C), we noted a reduction in the polysome peak of SMA profiles, suggestive of translation defects. To quantify this effect, we determined the fraction of ribosomes engaged on polysomes (FRP) at different stages of disease (Experimental Procedures and Figures 1F and 1G). This value provides an estimate of the translation status/activity of tissues and cells, describing the engagement of ribosomes on RNAs in polysomes and/or the recruitment of mRNAs on polysomes for translation (Anand et al., 2003, Brina et al., 2015, Ortiz and Kinzy, 2005). We compared FRP values in different tissues of SMA mice at different stages of disease with age-matched controls, finding no detectable changes at pre-symptomatic stages in any tissue. Strikingly, however, we observed a strong decrease in FRP in SMA spinal cord from symptomatic mice (Figure 1G). In addition, we noted a significant decrease at early- and late-symptomatic stages in SMA brain (Figure 1F), indicating that translation can be affected early in the disease process. A decrease in kidney but no change in liver (Figures S1C–S1E) suggests that translation defects display tissue specificity, with the most pathologically affected tissues in SMA showing the greatest magnitude of change.

To investigate the temporal nature of translation changes, we compared the extent of translation impairment with established SMA phenotypic disease readouts: body weight (Hsieh-Li et al., 2000) and righting time (Le et al., 2005). SMA mice were easily discernible from control littermates at early- and late-symptomatic stages, as demonstrated by decreased body weight and increased righting time (Data S1). In contrast, at pre-symptomatic stages, SMA and control mice were indistinguishable. In control and pre-symptomatic SMA mice, normal body weight and short righting times were associated with high FRP values in brain (Figure 1H) and spinal cord (Figure 1I). In contrast, in early- and late-symptomatic SMA mice, lower FRP values corresponded with the appearance of disease symptoms. The relationship between lower FRP values and lower relative weight (Figure 1H and 1I, left panels) and longer time to right (Figures 1H and 1I, right panels, and Figures S1E and S1F) confirmed that translation defects in SMA are associated with established phenotypic readouts of disease progression. Thus, FRP values reflect translation defects downstream of SMN depletion that co-occur with early phenotypic changes in SMA.

### Translation Defects Are SMN Dependent

**Figure 2:**
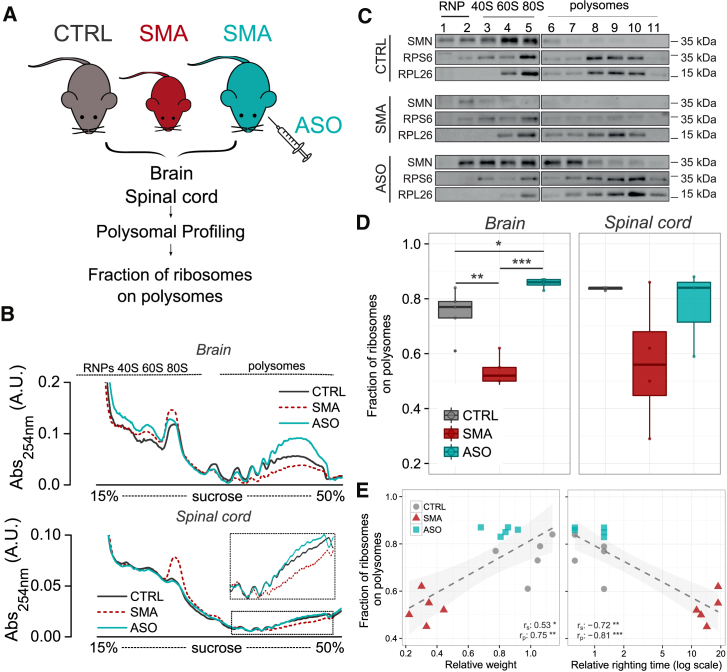
Treatment with ASO Recovers SMN Expression and Translation Defects in SMA Tissues (A) Experimental design. (B) Representative polysomal profiles obtained from brains (top) and spinal cords (bottom) in each experimental group. (C) Representative polysomal profiles obtained from CTRL, SMA, and SMA-ASO mouse brains (late symptomatic) and corresponding co-sedimentation profiles from each experimental group for SMN, RPS6, and RPL26. (D) FRP from CTRL, late-symptomatic SMA and SMA-ASO brains (left) and spinal cords (right) (CTRL, n = 5; SMA, n = 5; ASO, n = 5, ^∗^p < 0.05, ^∗∗^p < 0.01, ^∗∗∗^p < 0.001, two-tailed t test). (E) Relationship between body weight (left) or righting time (right) and the corresponding FRP, obtained from CTRL, SMA, and ASO-treated mouse brains. Each point corresponds to one mouse. Spearman and Pearson correlations are indicated. (^∗^p < 0.05, ^∗∗^p < 0.01, ^∗∗∗^p < 0.001, correlation test). See also Figure S2.

Having demonstrated a decrease in the fraction of ribosomes on polysomes in SMA and the association of translation impairment with disease severity, we next wanted to establish whether these changes were directly dependent upon SMN protein expression. To assess this, we investigated polysome profiles of SMA mice treated with an established antisense oligonucleotide (ASO) that leads to restoration of SMN levels (Hua et al., 2011, Zhou et al., 2013). Mice received an intravenous injection of a 25-mer morpholino ASO (PMO25) that targets the *SMN2* intron 7 splicing silencer N1. As expected, this treatment increased levels of *SMN2* exon 7 inclusion in the central nervous system (CNS) (Zhou et al., 2013). After polysomal profiling (Figure 2A), we determined the FRP in brain and spinal cord of control, SMA, and ASO-treated SMA mice (Figure 2B). The increase in SMN protein levels restored SMN on polysomes (Figure 2C) and robustly rescued disease symptoms compared with untreated SMA mice (Figures S2A and S2B). As previously, loss of SMN from polysomes in late-symptomatic SMA mice was associated with a significantly decreased FRP in brain and spinal cord compared to littermate controls (Figure 2D). In contrast, ASO treatment not only restored the amount of SMN but actually increased levels compared to controls. This resulted in restoration of the amount of SMN on polysomes and the fraction of ribosomes in polysomes (Figure 2D). Moreover, FRP values after ASO treatment correlated with normal weight and righting times (Figures 2E, S2C, and S2D).

Thus, these results demonstrate that translational defects observed in SMA can be reversed by restoring SMN.

### Translation Impairment Is Cell Autonomous

Whether the defects in ribosome engagement on polysomes detailed above are a major driver of functional impairment of translation and/or cell autonomous remained to be determined.

**Figure 3:**
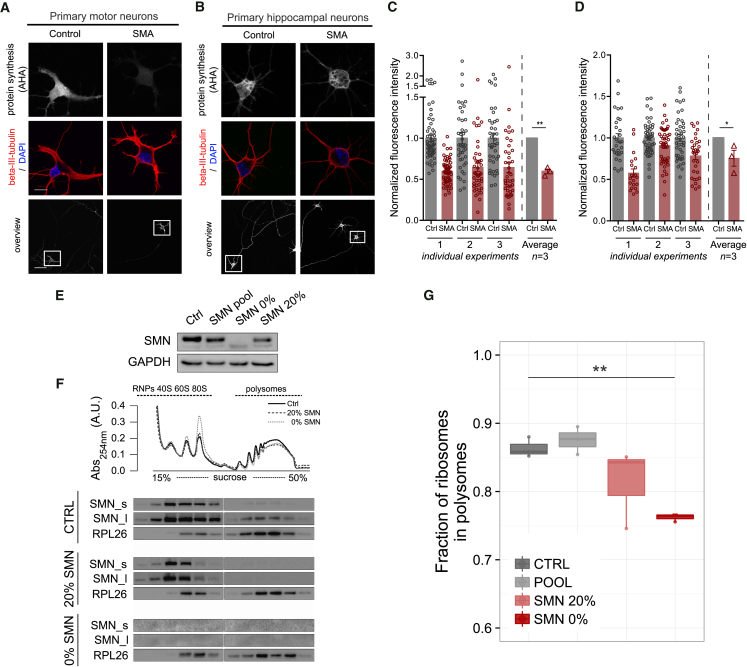
Translation Defects are Cell Autonomous and Dependent on SMN Loss (A and B) Primary motor neurons (A) and primary hippocampal neurons (B) from control and SMA embryos were stained to reveal overall morphology (beta-III-tubulin, red) and nuclear integrity (DAPI, blue). Protein synthesis was visualized by labelling newly synthesized proteins with L-azidohomoalanine (AHA, gray scale, scale bars: 50 μm (overview), 10 μm (cell bodies; beta-III-tubulin/DAPI and AHA). DAPI, 4’,6-diamindion-2-phenylindole). (C) AHA fluorescence intensity values in individual primary motor neurons in three independent preparations: SMA (n = 43, 41, and 56) and control (n = 42, 40, and 56). (D) AHA fluorescence intensity values in individual primary hippocampal neurons in three independent preparations: SMA (n = 29, 50, and 48) and control (n = 20, 48, and 32) (^∗∗^p < 0.01, ^∗^p < 0.05, Student’s t test; error bars ± SEM.). (E) SMN levels in NSC-34 native (CTRL), the pool of cells expressing different levels of SMN (POOL), and two specific clones expressing 20% and 0% of SMN. (F) Representative polysomal profiles from NSC-34 native (CTRL) and the two clones expressing 20% and 0% of SMN. Co-sedimentation profiles of SMN and RPL26 are shown. The signal of SMN is shown for short (SMN_s) and long (SMN_l) exposure times of acquisition. (G) Comparison between the FRP in NSC-34 native and expressing 20% and 0% of SMN (CTRL: n = 3; 20%: n = 3; 0%: n = 3). Significant decreases were identified with one-tailed t test (^∗∗^p < 0.01). See also Figure S3.

We therefore cultured primary motor neurons (the main affected neuronal cell type in SMA [Powis and Gillingwater, 2016]) and primary hippocampal neurons (a more modestly affected cell type [Wishart et al., 2010]) from SMA and control mice. We measured *de novo* protein synthesis levels using an established metabolic labelling technique based on incorporating the methionine-homologue L-azidohomoalanine (AHA) into newly synthesized proteins (Dieterich et al., 2010). When primary motor neurons were treated with anisomycin, an inhibitor of translation, the AHA fluorescence signal decreased to background levels (Figures S3A–S3C) while, when applying AHA metabolic labelling to primary SMA-derived motor and hippocampal neurons (Figures 3A and 3B), we observed a marked decrease in fluorescence: ∼37% decrease in SMA versus control motor neuron cell bodies (Figure 3C). Similarly, fluorescence intensity in axon segments of primary motor neuron revealed an ∼ 39% decrease (Figure S3D). Comparable quantification in primary hippocampal neurons indicated a more modest decrease of ∼21% (Figure 3D). In line with these results and with previously observed pathological changes in the hippocampus and cortex of SMA mice (Wishart et al., 2010), we found a significant decrease in FRP in dissected cortex and hippocampus from late-symptomatic SMA mice, while no changes were detected in the cerebellum (Figure S3E). These results demonstrate that defects in ribosomal engagement on polysomes in whole tissues (e.g. spinal cord or brain) are restricted to specific anatomical regions and cell populations (e.g., motor and hippocampal neurons), with the most robust defects in *de novo* protein synthesis observed in the primary affected cell type in SMA, the motor neuron.

Next, to study the role of SMN in inducing the observed translation defects, we developed motor-neuron-like cell lines (NSC-34) expressing SMN at defined levels using CRISPR/Cas9 technology. We isolated and expanded clones expressing 20% and 0% SMN (Figure 3E) and performed polysome profiling (Figure 3F) to determine the FRP. As seen in whole tissues, we observed translation defects that correlated directly with the relative level of SMN loss (Figure 3G).

Taken together, these data suggest that translation impairment is a cell-autonomous event in SMA that is regulated directly by SMN levels.

### Determining the *In Vivo* SMA Transcriptome and Translatome by Next-Generation Sequencing

**Figure 4:**
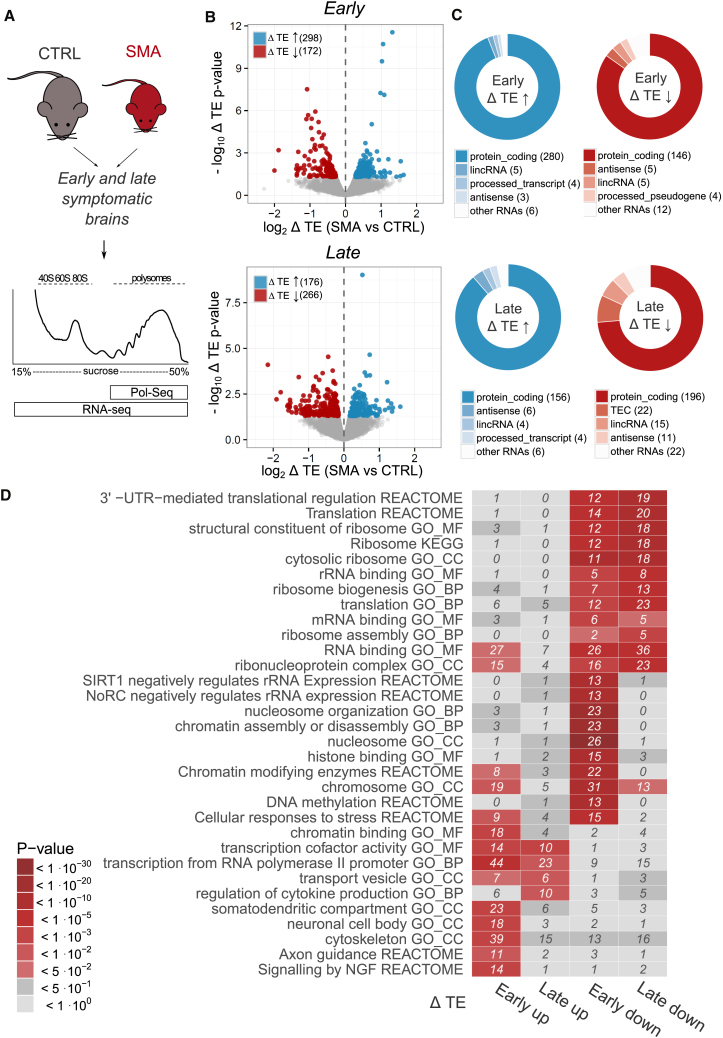
Identification of RNAs with Defective Translation Efficiency Reveals Functional Clusters of RNAs with Altered Translation in SMA (A) Experimental design for POL-seq and RNA-seq profiling on brains from late symptomatic SMA and control mice. (B) Volcano plot displaying translation efficiency variations (x axis) and associated p values (y axis) in early symptomatic (top) and late symptomatic (bottom) SMA brains compared to controls. Genes with statistically significant variations in TE are labelled according to the direction of the change: up (blue) or down (red) regulation in SMA. (C) RNA subtypes of genes with significantly altered TE in early- (top) and late-symptomatic (bottom) SMA mice. (D) Heatmap with top enriched terms (from Gene Ontology) and pathways (from KEGG and Reactome). Enrichment analysis was performed on genes with significant changes of TE in early- and late-symptomatic SMA brains. The number of genes contributing to the enrichment is indicated in each tile. See also Figure S4 and Data S2 and S3.

We used next-generation sequencing (NGS) to identify and quantify, in parallel, the total cytoplasmic RNA (RNA-seq) and the RNAs associated with polysomes (POL-seq). We extracted total cytoplasmic and polysomal RNAs from pooled sucrose fractions from control, early-, and late-symptomatic SMA mice (Figure 4A) to simultaneously identify variations in RNA populations at both *translatome* (RNAs engaged with polysomes) and *transcriptome* (steady-state cytoplasmic RNAs) levels (Data S2). Transcriptome and translatome changes were moderately correlated, with an overlap of approximately one-third between differentially expressed genes (DEGs, 27% in early-symptomatic, 39% in late-symptomatic mice, see Figure S4A). To confirm the data, we chose a set of nine differentially expressed genes across early- and late-symptomatic stages for validation by qPCR. We found a high concordance between the NGS and qPCR approaches at both stages (r^2^ =0.77 for early-symptomatic stage and r^2^ =0.99 for late-symptomatic stage [Figure S4B]).

To understand whether variations identified at early- and late-symptomatic stages and at translational and transcriptional levels were also hallmarks of pre-symptomatic disease stages, we determined an expression time course for transcripts changed at both levels previously selected for qPCR validation (Figure S4C). We found that some transcripts were already differentially expressed at pre-symptomatic stages, indicating early pathogenic changes. Next, we determined the expression of the same set of transcripts in RNA isolated from spinal cord. Most changes could be detected not only in brain but also in spinal cord (Figure S4D). Moreover, these changes were reversible, as ASO treatment that restored SMN levels (Figure 2) normalized the expression at both levels (Figure S4E). Finally, to confirm that at least some of the changes identified in SMA on polysomes resulted in corresponding changes at the protein level, we measured levels of UBA6 and RGS5. Both proteins showed a decrease in expression that matched changes at the RNA level (Figure S5). Thus, transcripts identified as differentially expressed in SMA by RNA-seq and POL-seq reflect early changes during disease progression, including variations in the spinal cord and at the protein level. Moreover, these changes are reversible by ASO treatment restoring SMN expression.

### Alterations in Translation Efficiency Highlight Defects in Ribosome Biology in SMA

Having determined, in parallel, the transcriptome (RNA-seq) and translatome (POL-seq), we next calculated the ratio between POL-seq and RNA-seq data for each transcript, exploring changes in the equilibrium between transcription and translation in SMA. This approach also allowed us to obtain a transcriptome-wide profile of individual genes that were affected by the widespread translation defects we reported above. We generated a comprehensive catalogue of changes in translation efficiencies (TEs) (Tebaldi et al., 2014, Xiao et al., 2016) of individual coding and non-coding RNA transcripts in control and SMA tissue at both early- and late-symptomatic stages (Figure 4B and Data S3). This catalogue comprises 470 and 442 transcripts with significantly altered TE at early- and late-symptomatic stages, respectively. Interestingly, most RNAs with altered TE are protein coding, but a small fraction of these RNAs is noncoding (Figure 4C). Genes with significantly altered TE were mostly different from DEGs identified from the separated analysis of transcriptome and translatome variations (Figure S4F).

To gain insight into the molecular processes affected by changes in TE, we performed Gene Ontology and pathway enrichment analyses on transcripts with altered TE values (Figure 4D and Data S3). When focusing on transcripts with reduced levels of TE at early stages of disease, two major cellular processes emerged: nucleosome/chromatin and ribosome/translation. Interestingly, however, the ribosome/translation category was the only one robustly conserved at the late stage (Figure 4D). This suggests that defects in ribosome/translation are an early event following SMN depletion that persist into late stages of SMA. Notably, enrichment of translation/ribosome terms was also specific for translatome DEGs in early symptomatic mice (Figure S4G). This suggests that TE variations for these genes are primarily driven by changes in their polysomal RNA levels in early symptomatic mice.

**Figure 5:**
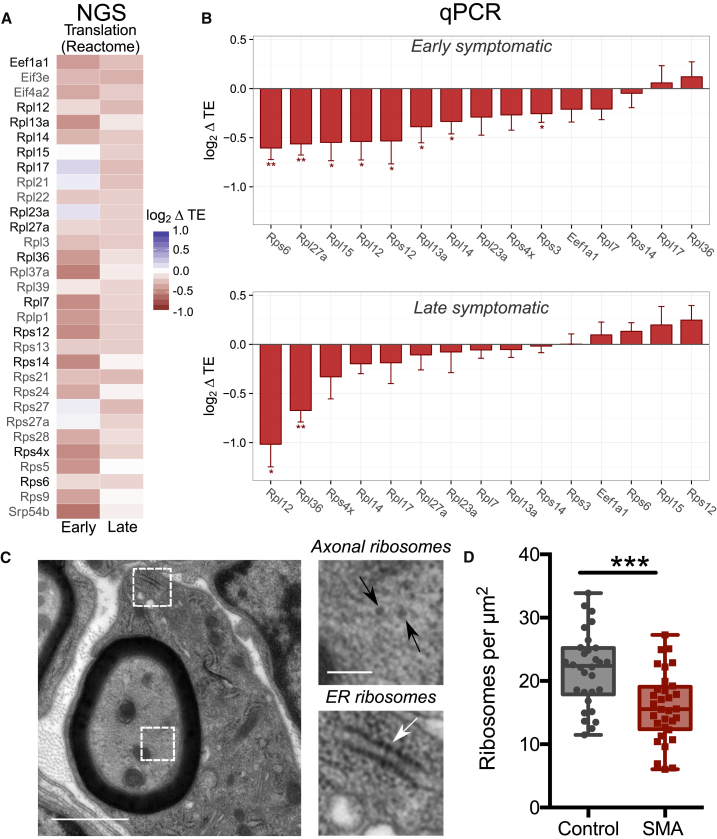
Translation Alterations in SMA Reveal Links between SMN and Ribosome Biology (A) Heatmap displaying all genes with altered translation efficiency (ΔTE) in SMA brains and annotated under the “Translation” Reactome pathway, significantly enriched in Figure 4D. The majority of genes belong to the family of ribosomal proteins. Genes further analyzed by qPCR are highlighted in black. (B) qPCR-derived variations of translation efficiency for ribosomal proteins and one elongation factor from (A) in early (top) and late (bottom) symptomatic mouse brains. Mean value ± SEM is shown; three to four biological replicates and two to six technical replicates; all genes were normalized to the geometric mean of actin and cyclophilin a; one-tailed t test; ^∗^p < 0.05, ^∗∗^p < 0.01, ^∗∗∗^p < 0.001. (C) Representative electron micrograph of a large diameter (motor) axon in the intercostal nerve from a P5 SMA mouse (black arrowheads: axonal ribosomes; white arrowhead, ER ribosomes). (D) Counts of axonal ribosomes revealed a decrease in the density of ribosomes in SMA mouse axons compared to controls (n = 30 axon profiles; N = 3 mice per genotype; ^∗∗∗^p < 0.001, two-tailed t test). See also Figure S5.

A large majority of transcripts with a significantly reduced TE and associated with “translation”-related processes were ribosomal proteins, translation initiation and elongation factors, and proteins involved in ribosome biogenesis (Figure 5A). We quantified individual transcript levels for 14 ribosomal genes and one translation factor by qPCR and confirmed that the expression of numerous transcripts involved in ribosome biogenesis (Jakovljevic et al., 2012, Landowski et al., 2013, Lo et al., 2009) is affected in SMA, both at early- and late-symptomatic stages (Figure 5B). Moreover, western blot analysis confirmed that reduced TE of ribosomal proteins RPS4X and RPS6 leads to reduced protein levels at late-symptomatic stages (Figure S5), in line with our hypothesis that defects in ribosome abundance could underlie the decreased FRP (Figure 1) and other translation defects observed in SMA.

Finally, to establish whether downregulation of translation-related transcripts leads to downstream functional changes in ribosome biology *in vivo*, we performed ultrastructural analyses of ribosomes in motor axon profiles from intercostal nerves isolated from symptomatic severe SMA mice and littermate controls (Hunter et al., 2016). As expected, transmission electron microscopy revealed prominent axonal ribosomes in both control and SMA mice (Figure 5C). Importantly, quantitative analyses of axonal ribosomal density revealed a 27% decrease in the number of axonal ribosomes in intercostal nerves from SMA mice versus controls (Figure 5D).

Thus, cellular processes identified through genome-wide analysis of TE in SMA allowed us to pinpoint hallmarks of SMA *in vivo*, demonstrating that SMN plays a role in translation and ribosome biology.

## Discussion

Increasing evidence suggests a role for translation defects in the pathogenesis of neurodegenerative diseases. Ribosomal defects have been documented in conditions such as Alzheimer’s disease, Huntington’s disease, Parkinson’s disease, and frontotemporal dementia (Ding et al., 2005, Lee et al., 2011, Vilotti et al., 2012). Here, we found that SMN protein is an important mediator of translation in the nervous system during SMA. We employed a range of methods to reveal a role for SMN in regulating translation *in vitro* and *in vivo*, using NGS to determine widespread changes occurring in the translatome in SMA and to generate a unique catalogue of RNAs with altered translation efficiency. We established that SMN depletion leads to an early and robust impairment of translation-related transcripts in SMA and to consequent defects in ribosomal biology. In addition, we demonstrated that translation defects are cell autonomous and can be rescued by therapies increasing SMN protein levels, suggesting that defects in translation and ribosome biology are core pathological hallmarks of SMA. In this context, our findings provide *in vivo* evidence supporting the hypothesized physiological role of SMN in the localization of the translation machinery and translation of specific transcripts proposed previously by *in vitro* studies (Fallini et al., 2011, Fallini et al., 2016, Gabanella et al., 2016, Sanchez et al., 2013).

One longstanding issue in several neurodegenerative diseases is the lack of a robust explanation for the cell-type specificity that results from defects in ubiquitously expressed proteins. Indeed, this is also a feature of SMA following ubiquitous depletion of SMN protein (Hamilton and Gillingwater, 2013). Although spinal motor neurons are the most prominently affected cell type, numerous other cell types and tissues can also be affected in SMA, both in patients and animal models (e.g., Shababi et al., 2010, Somers et al., 2016). Translation defects we report here were notably most pronounced in brain and spinal cord, with more modest defects observed in kidney and no defects in the liver. These findings support a threshold model for the requirement of SMN, where different tissues and cell types require different minimal levels of SMN and are thus differentially susceptible to reduced levels of SMN (Sleigh et al., 2011). Our catalogue of RNA defects suggests that translation and ribosome biology are major downstream mediators of the effects of low levels of SMN across different tissues in SMA.

Interestingly, our parallel transcriptome and translatome analyses revealed that one-third of the transcriptional changes that occur in SMA are directly reflected in translation changes of the same transcripts. Indeed, large changes in the transcriptome driven by alterations in functional translation or ribosome biogenesis can be primarily caused by feedback mechanisms at the transcriptional level (Santagata et al., 2013), pointing toward translation as possible *primum movens* of transcriptional changes. The fact that altered splicing alone cannot explain SMA pathogenesis (Bäumer et al., 2009, Shpargel and Matera, 2005) and that very few transcriptional changes have been detected in pre-symptomatic SMA tissues (Doktor et al., 2016) emphasizes that SMA pathogenesis is likely to be multifactorial. Therefore, further hypotheses are required to better understand the complex mechanistic role of SMN in controlling RNA metabolism in the cytoplasm, especially with respect to the translation machinery in general. Given the well-known nuclear role of SMN, it is tempting to speculate that SMN may coordinate, together with other proteins, the fine equilibrium between RNA synthesis, nuclear export, transport, translation, and ribosome biogenesis, whose balance is likely to be particularly critical for neurons. In agreement with this hypothesis, SMN can localize to the nucleolus, where it interacts with U3 snoRNA involved in processing of ribosomal RNA (rRNA) (Wehner et al., 2002). Among the lncRNAs showing defects in SMA, several were found to associate with polysomes, supporting the hypothesis that lncRNAs are likely to influence the activity of polysomes (Carlevaro-Fita et al., 2016, Ingolia et al., 2011). Interestingly, among those RNAs with altered TE, we identified several snoRNAs involved in ribosome biogenesis. Similarly, the characterization of the SMN interactome identified likely interactions between the SMN complex and numerous ribosomal proteins, as well as the translation elongation factor eEF1A1 (Fuller et al., 2010). It is possible, therefore, that low levels of SMN impact the proper production of ribosomes in SMA, especially in the most affected cells and tissues. Interestingly, a recent study showed that ribosomes with different subsets of ribosomal proteins control pools of mRNAs with specific functions in mESCs (Shi et al., 2017). This raises another intriguing hypothesis: SMN might induce, indirectly or directly, changes in the ribo-proteome, impacting specialized ribosomes and changing their selectivity for translational regulation of subpopulations of axonal-specific mRNAs.

Taken together with findings from other neurodegenerative conditions, our study adds experimental weight to the hypothesis that translation represents a common molecular “hub” in disease pathogenesis. Indeed, recent studies of amyotrophic lateral sclerosis (ALS) have highlighted a role for translation defects. For example, one major pathological protein in ALS, TDP43, has been shown to regulate the translation of specific transcripts (Coyne et al., 2014). Similarly, ALS-associated protein aggregates affect the function of proteins that play an important role in regulating translation, such as FMRP (Blokhuis et al., 2016, Coyne et al., 2015), and mislocalization of axonal ribosomes occurs in ALS patients (Verheijen et al., 2014). It is interesting to note that many proteins that have been implicated in regulating translation and are associated with neurodegenerative diseases contribute to larger RNA-protein complexes (Blokhuis et al., 2016, Groen et al., 2013, Liu-Yesucevitz et al., 2011, Piazzon et al., 2008). This complicates the process of separating causative from secondary changes in disease, as likely functional redundancy in these complexes makes it challenging to detail true pathogenic changes. The use of powerful emerging technologies facilitating genetic tagging of ribosomes *in vivo* (e.g., Shigeoka et al., 2016), will likely play a major role in future investigations of the role of ribosome biology in motor neuron diseases such as SMA and ALS.

## Experimental Procedures

### Animal Models and ASO Treatment

“Taiwanese” (*Smn*^*−/−*^*; SMN2tg/0*) severe SMA mice (Powis and Gillingwater, 2016) on a congenic FVB background were generated from breeding pairs originally purchased from Jackson Labs. Phenotypically normal heterozygous (*Smn*^*−/+*^*;SMN2tg/0*) littermates were used as controls. For EM experiments, tissue had been banked from a “severe” SMA mouse colony (Hunter et al., 2014). Litters were genotyped using standard PCR protocols. Mice were housed in Edinburgh and UCL (London) under standard SPF conditions. All animal procedures and breeding were in accordance with University of Edinburgh and UCL institutional guidelines and under appropriate project and personal licenses granted by the UK Home Office.

ASO treatment was performed at UCL as previously described (Zhou et al., 2013). Disease progression of transgenic mice can vary between laboratories (Casellas, 2011). We therefore refer to time points at which tissue was collected as pre-, early- and late-symptomatic (Edinburgh: pre-symptomatic, P3; early symptomatic, P5; and late symptomatic, P7). Tissue from UCL was from late-symptomatic (P10) mice that were phenotypically similar to P7 mice in Edinburgh.

### Motor Performance Test

Mice were weighed daily. Motor performance was recorded using the righting reflex test (Passini et al., 2010, Wishart et al., 2014). Righting tests were performed in triplicate for each mouse and an average time calculated. If a mouse took longer than 30 s to right, the test was terminated.

### Tissue Isolation

Neonatal mice were euthanized by overdose of anesthetic before decapitation to remove the brain. Spinal cord dissection was performed by removing the spinal column and then using a needle and syringe to flush out the intact cord (Powis and Gillingwater, 2016). Peripheral tissues (kidney and liver) were subsequently dissected. All tissues were immediately snap-frozen on dry ice and stored at −80°C.

### Primary Neuronal Cultures

Primary motor neurons and hippocampal neurons were cultured as described previously (Van Battum et al., 2014, Blokhuis et al., 2016). All primary cultures were grown in an incubator at 37°C and 5% CO_2_.

### Labeling of Newly Synthesized Proteins in Primary Neurons

Novel protein synthesis was quantified using L-azidohomoalanine (AHA) and the Click-IT AHA kit (ThermoFisher Scientific) according to manufacturer’s recommendations. After 7 days in culture, cells were washed with PBS, and the medium was replaced with methionine-free medium, supplemented with B27, Glutamax, and AHA for 30 min. Cells were fixed in 4% PFA and permeabilized in 0.5% triton-X100 in PBS. The subsequent labelling of newly synthesized proteins was performed using Alexa Fluor 488. Nuclei were stained with DAPI. Cells were imaged on a Zeiss LSM710 confocal microscope, with identical settings used within each experiment. Images were analyzed in FIJI; the cell body was manually delineated and the mean fluorescence intensity measured. All analyses were performed with the operator blind to the genotype or treatment. For control experiments, neurons were pre-treated with 40 μM anisomycin for 30 min.

### Polysome Profiling

Cytoplasmic lysates from frozen mouse tissues were prepared as described previously (Lunelli et al., 2016). Cleared supernatants were loaded on a linear 15%–50% sucrose gradient and ultracentrifuged in a SW41Ti rotor (Beckman) for 1 hr and 40 min at 180,000 *g* at 4°C in a Beckman Optima LE-80K Ultracentrifuge. After ultracentrifugation, gradients were fractionated in 1 mL volume fractions with continuous monitoring absorbance at 254 nm using an ISCO UA-6 UV detector.

### Protein Extraction and Western Blot

Proteins were extracted from each sucrose fraction of the profile using the methanol/chloroform protocol (Wessel and Flügge, 1984) and solubilized in electrophoresis sample buffer (Santa Cruz Biotechnology) for the following SDS-PAGE and western blot analysis. SDS-PAGE was performed in a 12% gel or a 4%–12% gradient gel (Novex) and blotted on nitrocellulose or PVDF membranes. Western blots were performed using primary antibodies against RPL26 (1:2,000, Abcam), RPS6 (1:1,000, Cell Signalling), SMN (1:1,000, BD Transduction Laboratories), and the appropriate horseradish peroxidase (HRP) conjugated secondary antibodies (1:5,000, Santa Cruz Biotechnology) or the IRDye secondary antibodies (LICOR). Detection was performed using the ECL Prime Western Blotting Detection Reagent (Amersham) or when using infrared secondary antibodies, scanned on an Odyssey imager (LICOR) (Eaton et al., 2013).

### Fraction of Ribosomes on Polysomes

The FRP was calculated from polysomal profiles as the ratio between the area under the curve of polysomes and the area under the curve of polysomes plus the area of the 80S peak. At least three different polysomal profiles (min 3 – max 9) for each tissue (spinal cord or brain), physiological condition (heterozygous control or SMA), and disease stage (pre-, early or late symptomatic) were considered.

### Total and Polysomal RNA and Library Preparation

RNA was extracted from control and SMA brains, from ASO brains, and from control and SMA spinal cords according to Tebaldi et al. (2012). Profile fractions (total RNA) or polysomal fractions only (polysomal RNA) were pooled together. cDNA libraries were produced from 500 ng using the TruSeq Stranded Total RNA (Illumina) kit according to manufacturer's instructions.

### NGS Data Analysis

RNA-seq and POL-seq sequencing were performed with an Illumina HiSeq 2000 (*Mus musculus*, GPL13112). Fastq files were checked for quality control with FastQC. 100 bp reads generated from each sample were aligned to the mouse genome (GRCm38.p4) with Tophat (version 2.0.14), using the Gencode M6 transcript annotation as transcriptome guide. All programs were used with default settings. Mapped reads (ranging from 84% to 92% of total reads, Data S2) were assembled into transcripts guided by reference annotation (Gencode M6) with Cufflinks (version 2.2.1). Expression levels were quantified by Cufflinks with normalized FPKM (fragments per kilobase of exon per million mapped fragments). Differentially expressed genes and transcripts in RNA-seq and POL-seq samples were detected with CuffDiff (p < 0.05). Translation efficiency analysis was performed with Xtail (Xiao et al., 2016), and genes showing differential translation efficiency values were selected using a threshold on Xtail p value (< 0.05). Functional annotation of gene lists and enrichment analysis with Gene Ontology terms and KEGG or REACTOME pathways were performed with the clusterProfiler Bioconductor package.

### qPCR

The retrotranscription reaction was performed starting from 1 μg of RNA using the RevertAid First Strand cDNA synthesis kit (Thermo Scientific). qPCR was carried out using the CFX Connect Real-Time PCR Detection System (BioRad) using Kapa Syber Fast qPCR Mastermix (Kapa Biosystems). Primer sequences are provided in Data S4. Actin and cyclophilin A were used as reference genes. All reactions were performed in two to four biological replicates and two to six technical replicates. The obtained Ct values were used to calculate the fold change of each gene using the delta/delta Ct method (Livak and Schmittgen, 2001). The log2 delta TE was calculated as the difference between the fold change at the polysomal level and the fold change at the total level of the gene of interest.

### Electron Microscopy

Tissue was prepared, processed, and imaged using transmission electron microscopy, as described previously (Hunter et al., 2014).

### CRISPR/Cas9 Knockout NSC-34 Cells

Stable SMN knockout in NSC-34 cells was generated by CRISPR/Cas9 technology. NSC-34 motor neuron-like cell line was grown in Dulbecco’s modified Eagle’s medium (DMEM, Invitrogen), 10% FBS (GIBCO, Life Technologies), 2 mM L-Glutamine (GIBCO, Life Technologies), and 1% penicillin/streptomycin (GIBCO, Life Technologies) at 37°C in a 5% CO_2_ atmosphere. RNA guides were designed to target the exon 3 of mSMN (5′ CACCGAGTTGTGGCATTCTTCTTT 3′) and cloned in pX330-U6-Chimeric_BB-CBh-hSpCas9 plasmid (a gift from F. Zhang, Addgene plasmid 42230) implemented with a puromycin resistance gene (Cong et al., 2013). Cells were transfected using Lipofectamine 2000 (Life Technologies. Cells were selected using 5 μg/ml puromycin for 48 hr and clonal expanded. SMN expression levels were assessed by western blot.

### Statistical Methods

For hierarchical clustering based on FRP, relative weight and relative righting time data were first rescaled in order for each variable to have the same mean and standard deviation. The distance matrix was calculated with the “Euclidean” method. Clustering was performed with the “Ward.D” agglomeration method. Shifts in distribution mean values were tested with Student’s t test. All other statistical analyses were performed in Graphpad Prism, with individual statistical tests used indicated in the figure legends.

## Author Contributions

P.B., E.J.N.G., H.J.N., F.M.L., F.M., E.P., H.K.S., and T.H.G. performed all experiments; P.Z., P.B., and E.P. prepared the libraries; T.T. analyzed NGS data; V.P. produced the SMN CRISPR/Cas9 cell lines; H.Z. and F.M. performed ASO treatments in SMA mice; P.B., T.T., E.P., E.J.N.G., F.M., T.H.G., and G.V. prepared the figures; A.Q., T.H.G., and G.V. conceived experiments and directed the research; P.B., T.T., E.J.N.G., F.M.L., A.Q., T.H.G., and G.V. wrote the manuscript. All authors contributed during preparation, revision, and writing of the manuscript.

## Data and Software Availability

The accession number for the sequence reads reported in this paper is GEO: GSE102204.

## Supplemental Information

Document S1. Supplemental Experimental Procedures and Figures S1–S5

Data S1. Mice Phenotypic and FRP Data, Related to Figure 1

Data S2. Mapping Statistics and Complete Transcriptome and Translatome Data from RNA-Seq and POL-Seq Performed on Mice Brains at Early- and Late-Symptomatic SMA Stages, Related to Figure 4 Complete functional enrichment analyses for transcriptome and translatome DEGs.

Data S3. Complete Translation Efficiency Data from RNA-Seq and POL-Seq Performed on Mice Brains at Early- and Late-Symptomatic SMA Stages, Related to Figure 4 Complete functional enrichment analysis on transcripts with altered translation efficiency values in SMA mice brains.

Data S4. List of Primers Used for qPCR, Related to Experimental Procedures

Document S2. Article plus Supplemental Information

## References

[bib1] Anand M., Chakraburtty K., Marton M.J., Hinnebusch A.G., Kinzy T.G. (2003). Functional interactions between yeast translation eukaryotic elongation factor (eEF) 1A and eEF3. J. Biol. Chem..

[bib2] Bäumer D., Lee S., Nicholson G., Davies J.L., Parkinson N.J., Murray L.M., Gillingwater T.H., Ansorge O., Davies K.E., Talbot K. (2009). Alternative splicing events are a late feature of pathology in a mouse model of spinal muscular atrophy. PLoS Genet..

[bib3] Béchade C., Rostaing P., Cisterni C., Kalisch R., La Bella V., Pettmann B., Triller A. (1999). Subcellular distribution of survival motor neuron (SMN) protein: possible involvement in nucleocytoplasmic and dendritic transport. Eur. J. Neurosci..

[bib4] Blokhuis A.M., Koppers M., Groen E.J.N., van den Heuvel D.M.A., Dini Modigliani S., Anink J.J., Fumoto K., van Diggelen F., Snelting A., Sodaar P. (2016). Comparative interactomics analysis of different ALS-associated proteins identifies converging molecular pathways. Acta Neuropathol..

[bib6] Brina D., Miluzio A., Ricciardi S., Clarke K., Davidsen P.K., Viero G., Tebaldi T., Offenhäuser N., Rozman J., Rathkolb B. (2015). eIF6 coordinates insulin sensitivity and lipid metabolism by coupling translation to transcription. Nat. Commun..

[bib7] Brinegar A.E., Cooper T.A. (2016). Roles for RNA-binding proteins in development and disease. Brain Res..

[bib8] Burghes A.H.M., Beattie C.E. (2009). Spinal muscular atrophy: why do low levels of survival motor neuron protein make motor neurons sick?. Nat. Rev. Neurosci..

[bib9] Carlevaro-Fita J., Rahim A., Guigó R., Vardy L.A., Johnson R. (2016). Cytoplasmic long noncoding RNAs are frequently bound to and degraded at ribosomes in human cells. RNA.

[bib10] Casellas J. (2011). Inbred mouse strains and genetic stability: a review. Animal.

[bib11] Cong L., Ran F.A., Cox D., Lin S., Barretto R., Habib N., Hsu P.D., Wu X., Jiang W., Marraffini L.A. (2013). Multiplex genome engineering using CRISPR/Cas systems. Science.

[bib12] Cookson M.R. (2016). RNA-binding proteins implicated in neurodegenerative diseases. Wiley Interdiscip. Rev. RNA.

[bib13] Coyne A.N., Siddegowda B.B., Estes P.S., Johannesmeyer J., Kovalik T., Daniel S.G., Pearson A., Bowser R., Zarnescu D.C. (2014). Futsch/MAP1B mRNA is a translational target of TDP-43 and is neuroprotective in a Drosophila model of amyotrophic lateral sclerosis. J. Neurosci..

[bib14] Coyne A.N., Yamada S.B., Siddegowda B.B., Estes P.S., Zaepfel B.L., Johannesmeyer J.S., Lockwood D.B., Pham L.T., Hart M.P., Cassel J.A. (2015). Fragile X protein mitigates TDP-43 toxicity by remodeling RNA granules and restoring translation. Hum. Mol. Genet..

[bib15] Darnell J.C., Van Driesche S.J., Zhang C., Hung K.Y.S., Mele A., Fraser C.E., Stone E.F., Chen C., Fak J.J., Chi S.W. (2011). FMRP stalls ribosomal translocation on mRNAs linked to synaptic function and autism. Cell.

[bib16] Dieterich D.C., Hodas J.J.L., Gouzer G., Shadrin I.Y., Ngo J.T., Triller A., Tirrell D.A., Schuman E.M. (2010). In situ visualization and dynamics of newly synthesized proteins in rat hippocampal neurons. Nat. Neurosci..

[bib17] Ding Q., Markesbery W.R., Chen Q., Li F., Keller J.N. (2005). Ribosome dysfunction is an early event in Alzheimer’s disease. J. Neurosci..

[bib18] Doktor T.K., Hua Y., Andersen H.S., Brøner S., Liu Y.H., Wieckowska A., Dembic M., Bruun G.H., Krainer A.R., Andresen B.S. (2016). RNA-sequencing of a mouse-model of spinal muscular atrophy reveals tissue-wide changes in splicing of U12-dependent introns. Nucleic Acids Res..

[bib19] Donlin-Asp P.G., Bassell G.J., Rossoll W. (2016). A role for the survival of motor neuron protein in mRNP assembly and transport. Curr. Opin. Neurobiol..

[bib20] Dragon F., Gallagher J.E.G., Compagnone-Post P.A., Mitchell B.M., Porwancher K.A., Wehner K.A., Wormsley S., Settlage R.E., Shabanowitz J., Osheim Y. (2002). A large nucleolar U3 ribonucleoprotein required for 18S ribosomal RNA biogenesis. Nature.

[bib21] Eaton S.L., Roche S.L., Llavero Hurtado M., Oldknow K.J., Farquharson C., Gillingwater T.H., Wishart T.M. (2013). Total protein analysis as a reliable loading control for quantitative fluorescent Western blotting. PLoS ONE.

[bib22] Fallini C., Zhang H., Su Y., Silani V., Singer R.H., Rossoll W., Bassell G.J. (2011). The survival of motor neuron (SMN) protein interacts with the mRNA-binding protein HuD and regulates localization of poly(A) mRNA in primary motor neuron axons. J. Neurosci..

[bib23] Fallini C., Bassell G.J., Rossoll W. (2012). Spinal muscular atrophy: the role of SMN in axonal mRNA regulation. Brain Res..

[bib24] Fallini C., Donlin-Asp P.G., Rouanet J.P., Bassell G.J., Rossoll W. (2016). Deficiency of the Survival of Motor Neuron Protein Impairs mRNA Localization and Local Translation in the Growth Cone of Motor Neurons. J. Neurosci..

[bib25] Farrar M.A., Park S.B., Vucic S., Carey K.A., Turner B.J., Gillingwater T.H., Swoboda K.J., Kiernan M.C. (2017). Emerging therapies and challenges in spinal muscular atrophy. Ann. Neurol..

[bib26] Fuller H.R., Man N.T., Lam T., Thanh T., Keough R.A., Asperger A., Gonda T.J., Morris G.E. (2010). The SMN interactome includes Myb-binding protein 1a. J. Proteome Res..

[bib27] Gabanella F., Pisani C., Borreca A., Farioli-Vecchioli S., Ciotti M.T., Ingegnere T., Onori A., Ammassari-Teule M., Corbi N., Canu N. (2016). SMN affects membrane remodelling and anchoring of the protein synthesis machinery. J. Cell Sci..

[bib28] Groen E.J.N., Gillingwater T.H. (2015). UBA1: At the Crossroads of Ubiquitin Homeostasis and Neurodegeneration. Trends Mol. Med..

[bib29] Groen E.J.N., Fumoto K., Blokhuis A.M., Engelen-Lee J., Zhou Y., van den Heuvel D.M.A., Koppers M., van Diggelen F., van Heest J., Demmers J.A.A. (2013). ALS-associated mutations in FUS disrupt the axonal distribution and function of SMN. Hum. Mol. Genet..

[bib30] Hamilton G., Gillingwater T.H. (2013). Spinal muscular atrophy: going beyond the motor neuron. Trends Mol. Med..

[bib31] Hsieh-Li H.M., Chang J.G., Jong Y.J., Wu M.H., Wang N.M., Tsai C.H., Li H. (2000). A mouse model for spinal muscular atrophy. Nat. Genet..

[bib32] Hua Y., Sahashi K., Rigo F., Hung G., Horev G., Bennett C.F., Krainer A.R. (2011). Peripheral SMN restoration is essential for long-term rescue of a severe spinal muscular atrophy mouse model. Nature.

[bib33] Hunter G., Aghamaleky Sarvestany A., Roche S.L., Symes R.C., Gillingwater T.H. (2014). SMN-dependent intrinsic defects in Schwann cells in mouse models of spinal muscular atrophy. Hum. Mol. Genet..

[bib34] Hunter G., Powis R.A., Jones R.A., Groen E.J.N., Shorrock H.K., Lane F.M., Zheng Y., Sherman D.L., Brophy P.J., Gillingwater T.H. (2016). Restoration of SMN in Schwann cells reverses myelination defects and improves neuromuscular function in spinal muscular atrophy. Hum. Mol. Genet..

[bib35] Ingolia N.T., Lareau L.F., Weissman J.S. (2011). Ribosome profiling of mouse embryonic stem cells reveals the complexity and dynamics of mammalian proteomes. Cell.

[bib36] Jablonka S., Bandilla M., Wiese S., Bühler D., Wirth B., Sendtner M., Fischer U. (2001). Co-regulation of survival of motor neuron (SMN) protein and its interactor SIP1 during development and in spinal muscular atrophy. Hum. Mol. Genet..

[bib37] Jakovljevic J., Ohmayer U., Gamalinda M., Talkish J., Alexander L., Linnemann J., Milkereit P., Woolford J.L. (2012). Ribosomal proteins L7 and L8 function in concert with six A_3_ assembly factors to propagate assembly of domains I and II of 25S rRNA in yeast 60S ribosomal subunits. RNA.

[bib38] Jung H., Gkogkas C.G., Sonenberg N., Holt C.E. (2014). Remote control of gene function by local translation. Cell.

[bib39] King H.A., Gerber A.P. (2016). Translatome profiling: methods for genome-scale analysis of mRNA translation. Brief. Funct. Genomics.

[bib40] Landowski M., O’Donohue M.-F., Buros C., Ghazvinian R., Montel-Lehry N., Vlachos A., Sieff C.A., Newburger P.E., Niewiadomska E., Matysiak M. (2013). Novel deletion of RPL15 identified by array-comparative genomic hybridization in Diamond-Blackfan anemia. Hum. Genet..

[bib41] Le T.T., Pham L.T., Butchbach M.E.R., Zhang H.L., Monani U.R., Coovert D.D., Gavrilina T.O., Xing L., Bassell G.J., Burghes A.H.M. (2005). SMNDelta7, the major product of the centromeric survival motor neuron (SMN2) gene, extends survival in mice with spinal muscular atrophy and associates with full-length SMN. Hum. Mol. Genet..

[bib42] Lee J., Hwang Y.J., Boo J.H., Han D., Kwon O.K., Todorova K., Kowall N.W., Kim Y., Ryu H. (2011). Dysregulation of upstream binding factor-1 acetylation at K352 is linked to impaired ribosomal DNA transcription in Huntington’s disease. Cell Death Differ..

[bib43] Lefebvre S., Bürglen L., Reboullet S., Clermont O., Burlet P., Viollet L., Benichou B., Cruaud C., Millasseau P., Zeviani M. (1995). Identification and characterization of a spinal muscular atrophy-determining gene. Cell.

[bib44] Liu-Yesucevitz L., Bassell G.J., Gitler A.D., Hart A.C., Klann E., Richter J.D., Warren S.T., Wolozin B. (2011). Local RNA translation at the synapse and in disease. J. Neurosci..

[bib45] Livak K.J., Schmittgen T.D. (2001). Analysis of relative gene expression data using real-time quantitative PCR and the 2(-Delta Delta C(T)) Method. Methods.

[bib46] Lo K.-Y., Li Z., Wang F., Marcotte E.M., Johnson A.W. (2009). Ribosome stalk assembly requires the dual-specificity phosphatase Yvh1 for the exchange of Mrt4 with P0. J. Cell Biol..

[bib47] Lorson C.L., Hahnen E., Androphy E.J., Wirth B. (1999). A single nucleotide in the SMN gene regulates splicing and is responsible for spinal muscular atrophy. Proc. Natl. Acad. Sci. USA.

[bib48] Lunelli L., Bernabò P., Bolner A., Vaghi V., Marchioretto M., Viero G. (2016). Peering at Brain Polysomes with Atomic Force Microscopy. J. Vis. Exp..

[bib49] Lunn M.R., Wang C.H. (2008). Spinal muscular atrophy. Lancet.

[bib50] Ortiz P.A., Kinzy T.G. (2005). Dominant-negative mutant phenotypes and the regulation of translation elongation factor 2 levels in yeast. Nucleic Acids Res..

[bib51] Passini M.A., Bu J., Roskelley E.M., Richards A.M., Sardi S.P., O’Riordan C.R., Klinger K.W., Shihabuddin L.S., Cheng S.H. (2010). CNS-targeted gene therapy improves survival and motor function in a mouse model of spinal muscular atrophy. J. Clin. Invest..

[bib52] Piazzon N., Rage F., Schlotter F., Moine H., Branlant C., Massenet S. (2008). In vitro and in cellulo evidences for association of the survival of motor neuron complex with the fragile X mental retardation protein. J. Biol. Chem..

[bib53] Powis R.A., Gillingwater T.H. (2016). Selective loss of alpha motor neurons with sparing of gamma motor neurons and spinal cord cholinergic neurons in a mouse model of spinal muscular atrophy. J. Anat..

[bib54] Renton A.E., Chiò A., Traynor B.J. (2014). State of play in amyotrophic lateral sclerosis genetics. Nat. Neurosci..

[bib55] Rossoll W., Jablonka S., Andreassi C., Kröning A.-K., Karle K., Monani U.R., Sendtner M. (2003). Smn, the spinal muscular atrophy-determining gene product, modulates axon growth and localization of beta-actin mRNA in growth cones of motoneurons. J. Cell Biol..

[bib56] Sanchez G., Dury A.Y., Murray L.M., Biondi O., Tadesse H., El Fatimy R., Kothary R., Charbonnier F., Khandjian E.W., Côté J. (2013). A novel function for the survival motoneuron protein as a translational regulator. Hum. Mol. Genet..

[bib57] Santagata S., Mendillo M.L., Tang Y. -c., Subramanian A., Perley C.C., Roche S.P., Wong B., Narayan R., Kwon H., Koeva M. (2013). Tight coordination of protein translation and HSF1 activation supports the anabolic malignant state. Science.

[bib58] Sephton C.F., Yu G. (2015). The function of RNA-binding proteins at the synapse: implications for neurodegeneration. Cell. Mol. Life Sci..

[bib59] Shababi M., Habibi J., Yang H.T., Vale S.M., Sewell W.A., Lorson C.L. (2010). Cardiac defects contribute to the pathology of spinal muscular atrophy models. Hum. Mol. Genet..

[bib60] Shi Z., Fujii K., Kovary K.M., Genuth N.R., Röst H.L., Teruel M.N., Barna M. (2017). Heterogeneous Ribosomes Preferentially Translate Distinct Subpools of mRNAs Genome-wide. Mol. Cell.

[bib61] Shigeoka T., Jung H., Jung J., Turner-Bridger B., Ohk J., Lin J.Q., Amieux P.S., Holt C.E. (2016). Dynamic Axonal Translation in Developing and Mature Visual Circuits. Cell.

[bib62] Shpargel K.B., Matera A.G. (2005). Gemin proteins are required for efficient assembly of Sm-class ribonucleoproteins. Proc. Natl. Acad. Sci. USA.

[bib63] Sleigh J.N., Gillingwater T.H., Talbot K. (2011). The contribution of mouse models to understanding the pathogenesis of spinal muscular atrophy. Dis. Model. Mech..

[bib64] So B.R., Wan L., Zhang Z., Li P., Babiash E., Duan J., Younis I., Dreyfuss G. (2016). A U1 snRNP-specific assembly pathway reveals the SMN complex as a versatile hub for RNP exchange. Nat. Struct. Mol. Biol..

[bib65] Somers E., Lees R.D., Hoban K., Sleigh J.N., Zhou H., Muntoni F., Talbot K., Gillingwater T.H., Parson S.H. (2016). Vascular Defects and Spinal Cord Hypoxia in Spinal Muscular Atrophy. Ann. Neurol..

[bib66] Tebaldi T., Re A., Viero G., Pegoretti I., Passerini A., Blanzieri E., Quattrone A. (2012). Widespread uncoupling between transcriptome and translatome variations after a stimulus in mammalian cells. BMC Genomics.

[bib67] Tebaldi T., Dassi E., Kostoska G., Viero G., Quattrone A. (2014). tRanslatome: an R/Bioconductor package to portray translational control. Bioinformatics.

[bib68] Tiedje C., Ronkina N., Tehrani M., Dhamija S., Laass K., Holtmann H., Kotlyarov A., Gaestel M. (2012). The p38/MK2-driven exchange between tristetraprolin and HuR regulates AU-rich element-dependent translation. PLoS Genet..

[bib69] Tisdale S., Pellizzoni L. (2015). Disease mechanisms and therapeutic approaches in spinal muscular atrophy. J. Neurosci..

[bib70] Tisdale S., Lotti F., Saieva L., Van Meerbeke J.P., Crawford T.O., Sumner C.J., Mentis G.Z., Pellizzoni L. (2013). SMN is essential for the biogenesis of U7 small nuclear ribonucleoprotein and 3′-end formation of histone mRNAs. Cell Rep..

[bib71] Van Battum E.Y., Gunput R.A., Lemstra S., Groen E.J., Yu K.L., Adolfs Y., Zhou Y., Hoogenraad C.C., Yoshida Y., Schachner M. (2014). The intracellular redox protein MICAL-1 regulates the development of hippocampal mossy fibre connections. Nat. Commun..

[bib72] Verheijen M.H.G., Peviani M., Hendricusdottir R., Bell E.M., Lammens M., Smit A.B., Bendotti C., van Minnen J. (2014). Increased axonal ribosome numbers is an early event in the pathogenesis of amyotrophic lateral sclerosis. PLoS ONE.

[bib73] Vilotti S., Codrich M., Dal Ferro M., Pinto M., Ferrer I., Collavin L., Gustincich S., Zucchelli S. (2012). Parkinson’s disease DJ-1 L166P alters rRNA biogenesis by exclusion of TTRAP from the nucleolus and sequestration into cytoplasmic aggregates via TRAF6. PLoS ONE.

[bib74] Wehner K.A., Ayala L., Kim Y., Young P.J., Hosler B.A., Lorson C.L., Baserga S.J., Francis J.W. (2002). Survival motor neuron protein in the nucleolus of mammalian neurons. Brain Res..

[bib75] Wessel D., Flügge U.I. (1984). A method for the quantitative recovery of protein in dilute solution in the presence of detergents and lipids. Anal. Biochem..

[bib76] Wishart T.M., Huang J.P.-W., Murray L.M., Lamont D.J., Mutsaers C.A., Ross J., Geldsetzer P., Ansorge O., Talbot K., Parson S.H., Gillingwater T.H. (2010). SMN deficiency disrupts brain development in a mouse model of severe spinal muscular atrophy. Hum. Mol. Genet..

[bib77] Wishart T.M., Mutsaers C.A., Riessland M., Reimer M.M., Hunter G., Hannam M.L., Eaton S.L., Fuller H.R., Roche S.L., Somers E. (2014). Dysregulation of ubiquitin homeostasis and β-catenin signaling promote spinal muscular atrophy. J. Clin. Invest..

[bib78] Xiao Z., Zou Q., Liu Y., Yang X. (2016). Genome-wide assessment of differential translations with ribosome profiling data. Nat. Commun..

[bib79] Zhang Z., Lotti F., Dittmar K., Younis I., Wan L., Kasim M., Dreyfuss G. (2008). SMN deficiency causes tissue-specific perturbations in the repertoire of snRNAs and widespread defects in splicing. Cell.

[bib80] Zhou H., Janghra N., Mitrpant C., Dickinson R.L., Anthony K., Price L., Eperon I.C., Wilton S.D., Morgan J., Muntoni F. (2013). A novel morpholino oligomer targeting ISS-N1 improves rescue of severe spinal muscular atrophy transgenic mice. Hum. Gene Ther..

