# Supplementary material for: In Vivo Translatome Profiling in Spinal Muscular Atrophy Reveals a Role for SMN Protein in Ribosome Biology
Source: Cell Rep. 2017 Oct 24;21(4):953–65. doi: 10.1016/j.celrep.2017.10.010 (PMC5668566; doi:10.1016/j.celrep.2017.10.010)
Supplement: Document S1. Supplemental Experimental Procedures and Figures S1–S5 [file mmc1.pdf]

Cell Reports, Volume *21*

Supplemental Information

In Vivo Translatome Profiling in Spinal Muscular Atrophy Reveals a Role for SMN Protein in Ribosome Biology

Paola Bernabò, Toma Tebaldi, Ewout J.N. Groen, Fiona M. Lane, Elena Perenthaler, Francesca Mattedi, Helen J. Newbery, Haiyan Zhou, Paola Zuccotti, Valentina Potrich, Hannah K. Shorrock, Francesco Muntoni, Alessandro Quattrone, Thomas H. Gillingwater, and Gabriella Viero

Supplemental Figures and Legends

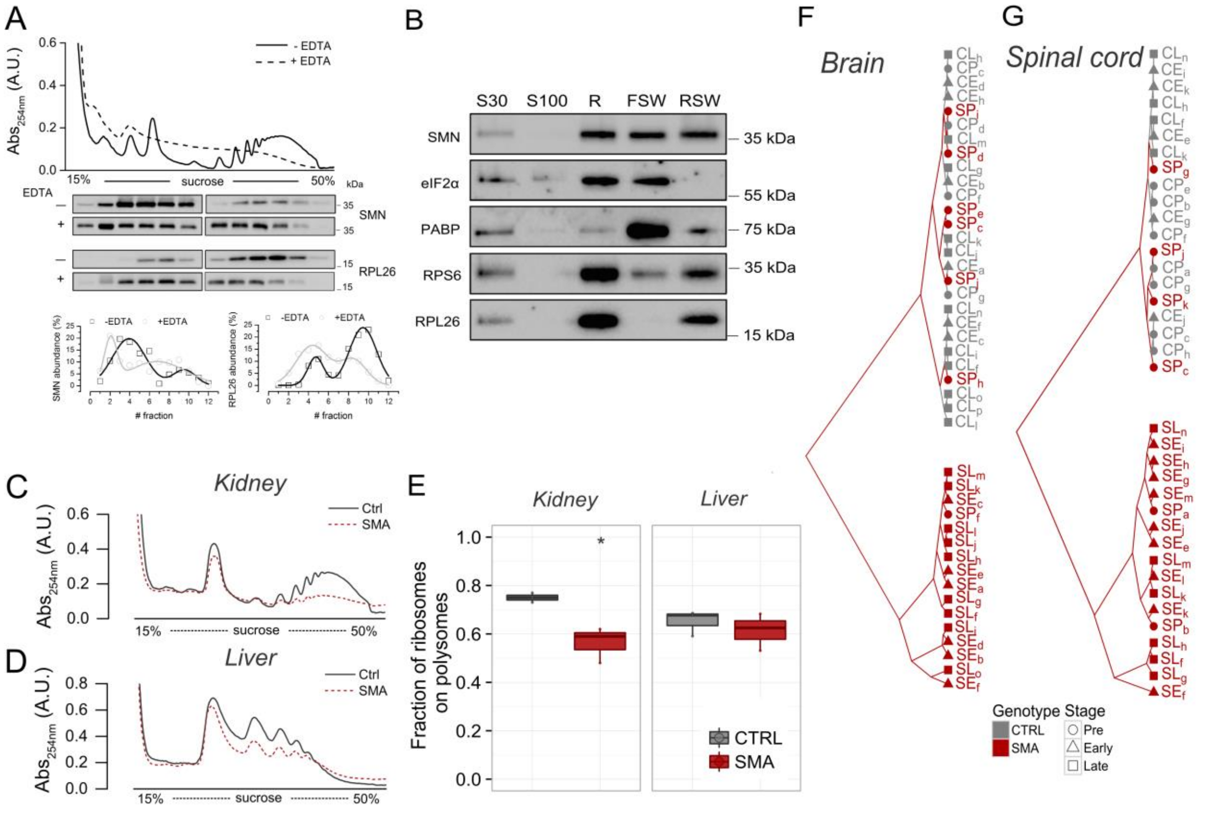

Figure S1. Related to Figure 1.
(A) SMN protein co-sediments with the translational machinery. NSC-34 nuclei/mitochondria free lysate was divided into two aliquots. One aliquot was directly fractionated on a 15–50% sucrose gradient (-EDTA, solid line). In parallel, the other half was treated with 8 mM EDTA (+ EDTA, dashed line). Fractions were collected and analyzed by western blot with antibodies against SMN and and the ribosomal L26 (RPL26) protein. **(B)** Subcellular fractionation and western blot analysis of S30 (input), S100 (soluble fraction), R (ribosomes fraction), SWR (salt washed ribosome fraction) RSW (pure ribosomes fraction) of NSC-34according to (Francisco-Velilla et al., 2016). The ribosomal proteins L26 and S6 were used as control of fractionation. The PolyA binding protein and the elongation factor eIF2a were used as controls for RNA-binding proteins interacting with polysomes though RNA-dependent interactions and for loosely ribosome- associated protein, respectively. **(C, D)** Representative polysomal profiles from control (Ctrl) and SMA kidneys and liver at late symptomatic stage. **(E)** Comparison between the fraction of ribosomes in polysomes in CTRL and SMA kidney and liver at late symptomatic stage (kidney: CTRL n=3, SMA n=3; liver: CTRL n=3, SMA n=3), * $P<0.05$, ** $P<0.01$, *** $P<0.001$, two-tailed t-test. (**F**, **G**) Dendrogram displaying hierarchical clustering of mice based on their relative weights, relative righting times and TE calculated from brain (**F**) or spinal cord (**G**). Each mouse is labelled according to genotype (C = Ctrl, S = SMA) and stage of disease (P = Pre, E = Early, L = Late). Individual mice are distinguished by subscript indices. Mice are colour-coded according to their genotype (Ctrl or SMA) and shape-labelled according to the time point at which they were sampled (pre (circle), early (triangle) or late (square)).

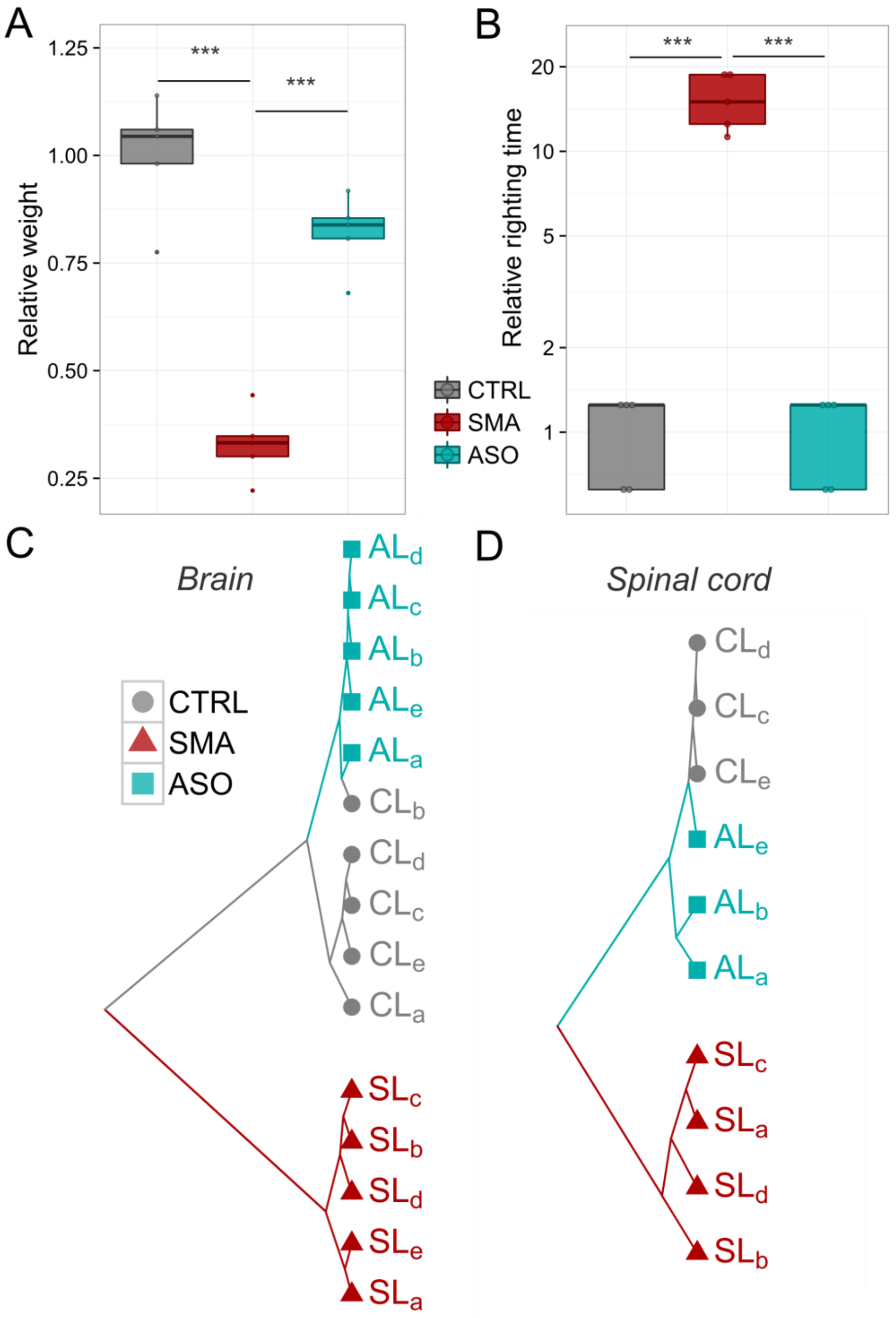

Figure S2. Related to Figure 2.
(**A**,**B**) Relative weights (**A**) and relative righting times (**B**) values plotted for Ctrl, SMA (late symptomatic) and ASO-treated SMA mice. Significant differences were identified with one-tailed Wilcoxon ranked-sum test (* P<0.05, ** P<0.01, *** P<0.001).(**C, D**) Clustering of changes in translation efficiency, body weight and righting time values distinguishes SMA mice from littermate controls and SMA mice treated with an ASO restoring SMN levels. Dendrogram displaying the hierarchical clustering of mice based on their relative weights, relative righting times and translation efficiencies calculated from brain (**C**) and spinal cord (**D**) extractions. Each mice is labelled according to the condition (C = Ctrl, S = SMA, A = ASO injected). Individual mice are distinguished by subscript indices.

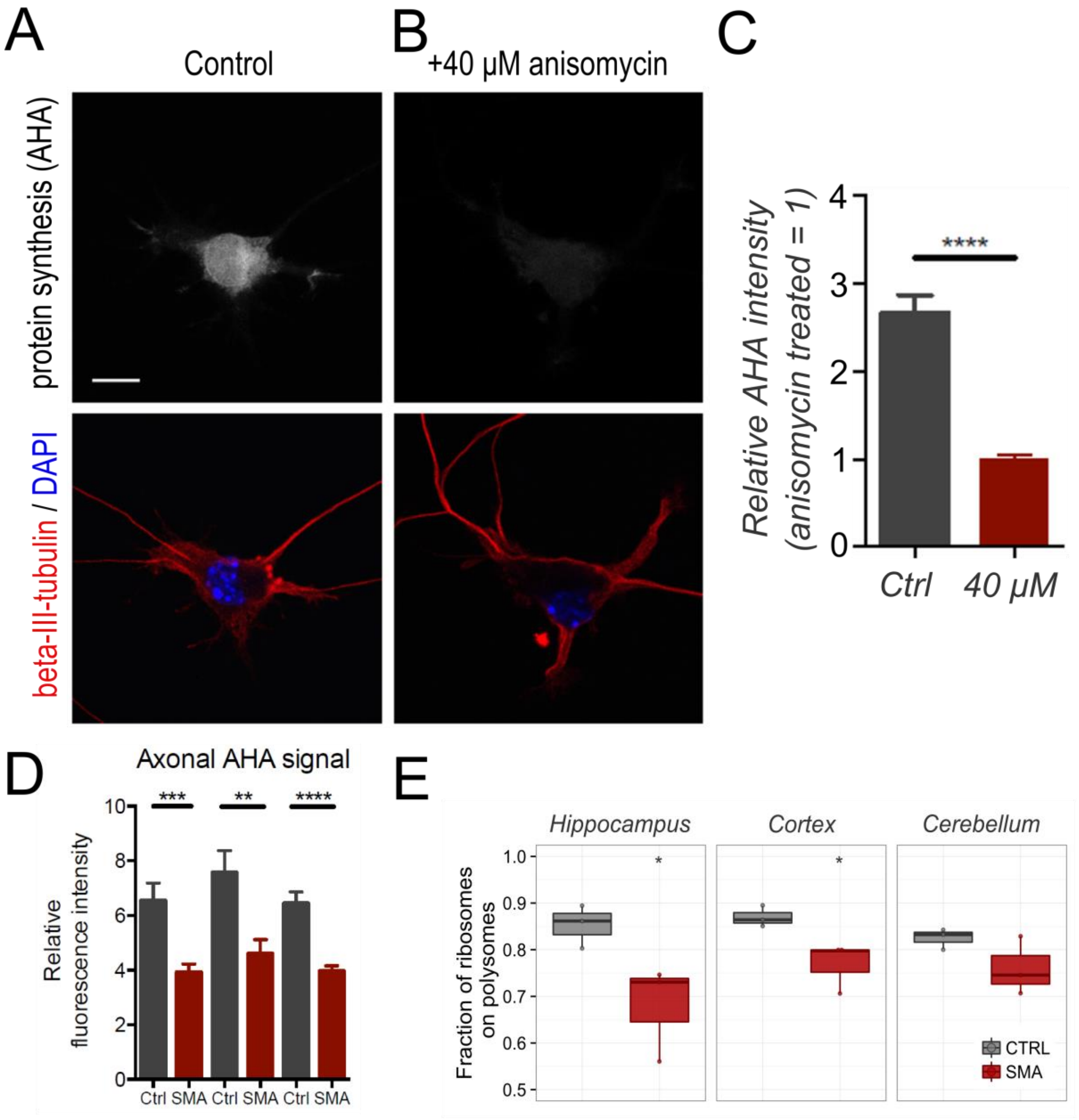

Figure S3. Related to Figure 3

(A-C) Anisomycin treatment confirms specificity of AHA labelling. Primary motor neurons were cultured from *smn+/-* control embryos and treated with 40 µM inhibitor of translation anisomycin (**B**) or DMSO (control, **A**). *De novo* protein synthesis was visualized by AHA labeling and performed as described above. Motor neurons were stained with beta-III-tubulin and DAPI to assess morphological and nuclear integrity (**A, C** lower panels). (**C**) Quantification of average AHA staining from 2 independent motor neuron preparations after anisomycin incubation intensity revealed a 62% decrease in staining intensity. (****P<0.0001, scale bar = 10 µm). (**D**) Protein synthesis is decreased in the axon of primary motor neurons. Protein synthesis was visualized by labelling newly synthesised proteins using L-azidohomoalanine (AHA) as in Figure 3. The fluorescence intensity of a 75 µm axonal segment from cultured primary motor neurons determined in ImageJ and determined for individual SMA and control neurons in each of 3 independent primary neuron preparations. Mean AHA signal was decreased by 40%, 39.2% and 38.6% for each of the respective experimental replicates. (**E**) Comparison between the fraction of ribosomes on polysomes in differentially affected brain regions from CTRL and SMA mouse brain regions: hippocampus (most affected), cortex and cerebellum (least affected) were analysed in late symptomatic mice (for each region 3 Ctrl and 3 SMA samples were analyzed). Significant reductions between CTRL and SMA brain regions were identified with one-tailed t-test (*P<0.05).

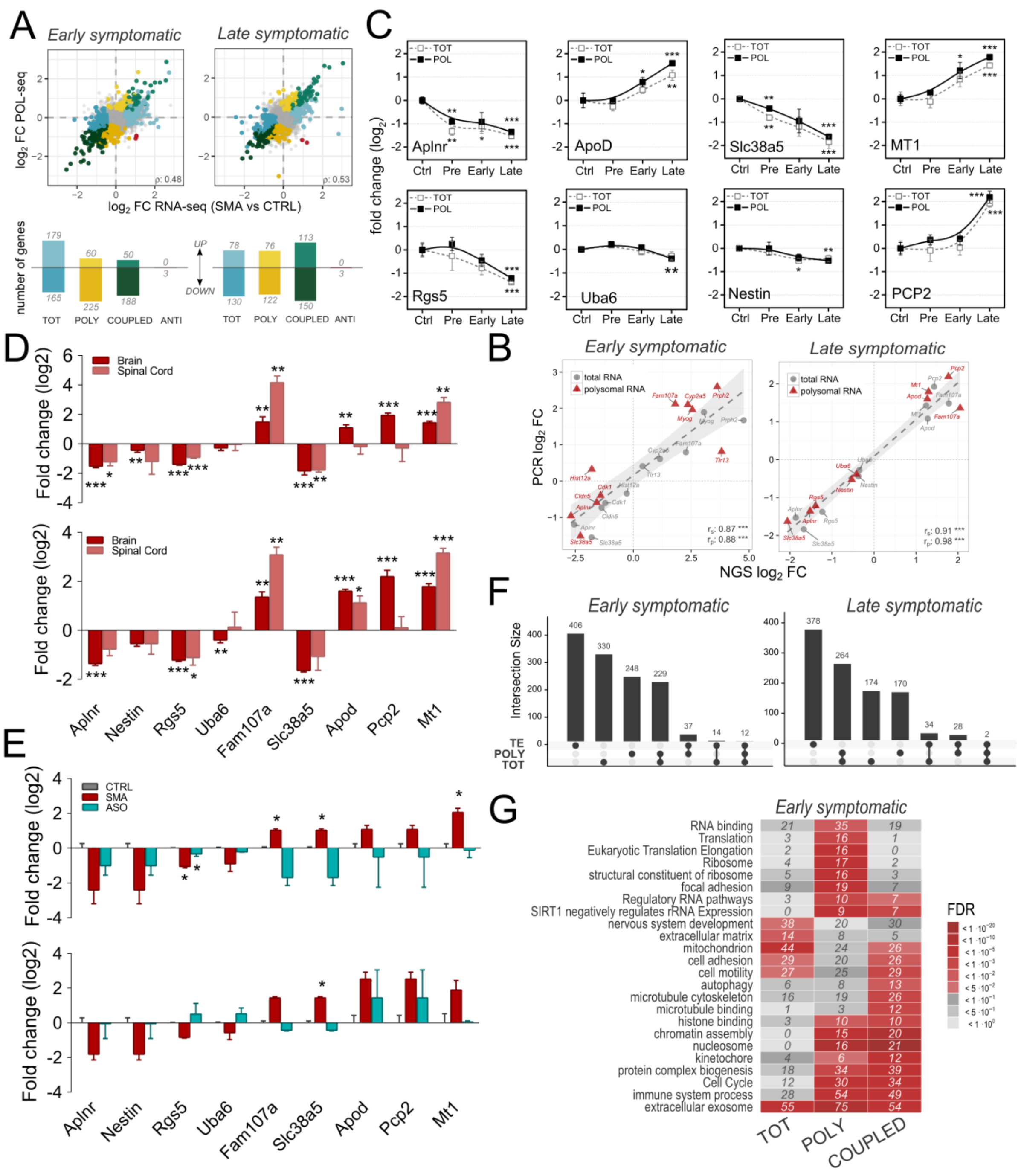

Figure S4. Related to Figure 4.

(A) Upper panel: scatterplots displaying translatome (y-axis) and transcriptome (x-axis) variations between SMA and control mice. The overall correlation between transcriptome and translatome variation is shown in the corner. Genes with statistically significant variations are colour labelled according to the orientation of the observed change (up or down regulation in SMA mice) and the level at which the change was observed: transcriptome only (TOT, in blue), translatome only (POLY, in yellow), both transcriptome and translatome with homodirectional changes (COUPLED, in green) or with antidirectional changes (ANTI, in red). Lower panel: barplots displaying the number of DEGs falling in each of the categories defined before. **(B)** Validation of POL-Seq and RNA-Seq by qPCR. The scatterplot compares NGS and validation by qPCR on a set of differentially expressed genes at early and late symptomatic stage of SMA. The significant coefficient of determination, as calculated by linear regression, demonstrates the high level of agreement between the two techniques. Both total RNA (circle) and polysomal RNA (triangle) data from brain tissues are shown. **(C)** Time-course of changes in levels of differentially expressed genes across three stages of disease with respect to the control brains at the transcriptional (TOT open symbols) and translation levels (POL, filled symbols). Analyses

were performed at pre-, early and late symptomatic stages for DEGs identified in late symptomatic tissue. All samples were normalized to the geometric mean value of actin and cyclophilin a. For each transcript the mean value ± SEM is shown (2-3 biological replicates and 2-4 technical replicates; * P<0.05, ** P<0.01, *** P<0.001, two-tailed unpaired t-test). **(D)** Comparison of differentially expressed genes between tissues (brain and spinal cord, late symptomatic) at transcriptional (upper panel) and translation levels (lower panel). All samples were normalized to the geometric mean value of actin and cyclophilin a. For each transcript the mean value ± SEM is shown (2-3 biological replicates and 2-4 technical replicates; * P<0.05, ** P<0.01, *** P<0.001, two-tailed unpaired t-test). **(E)** Comparison of differentially expressed genes after SMN-targeted treatment (Ctrl, SMA and ASO-treated brain, late symptomatic) at transcriptional (upper panel) and translation levels (lower panel). All samples were normalized to the geometric mean value of actin and cyclophilin a. For each transcript, the mean value ± SEM is shown (2 biological replicates and 4 technical replicates; * P<0.05, ** P<0.01, *** P<0.001, two-tailed unpaired t-test). (**F**) Barplots resuming intersection analysis performed on the lists of genes with significant variations in Translation efficiency (TE), translatome levels (POLY) and transcriptome levels (TOT). Plots were created with the UpSet visualization technique (Conway et al., 2017). (**G**) Heatmaps showing and comparing top enriched terms (from Gene Ontology) and pathways (from KEGG and Reactome). Enrichment analysis was performed on the lists of genes with significant changes exclusively in the transcriptome (TOT), exclusively in the translatome (POLY), concordantly in the transcriptome and the translatome (COUPLED). Significant enrichments are displayed in red shades. The number of genes contributing to the enrichment is indicated in each tile.

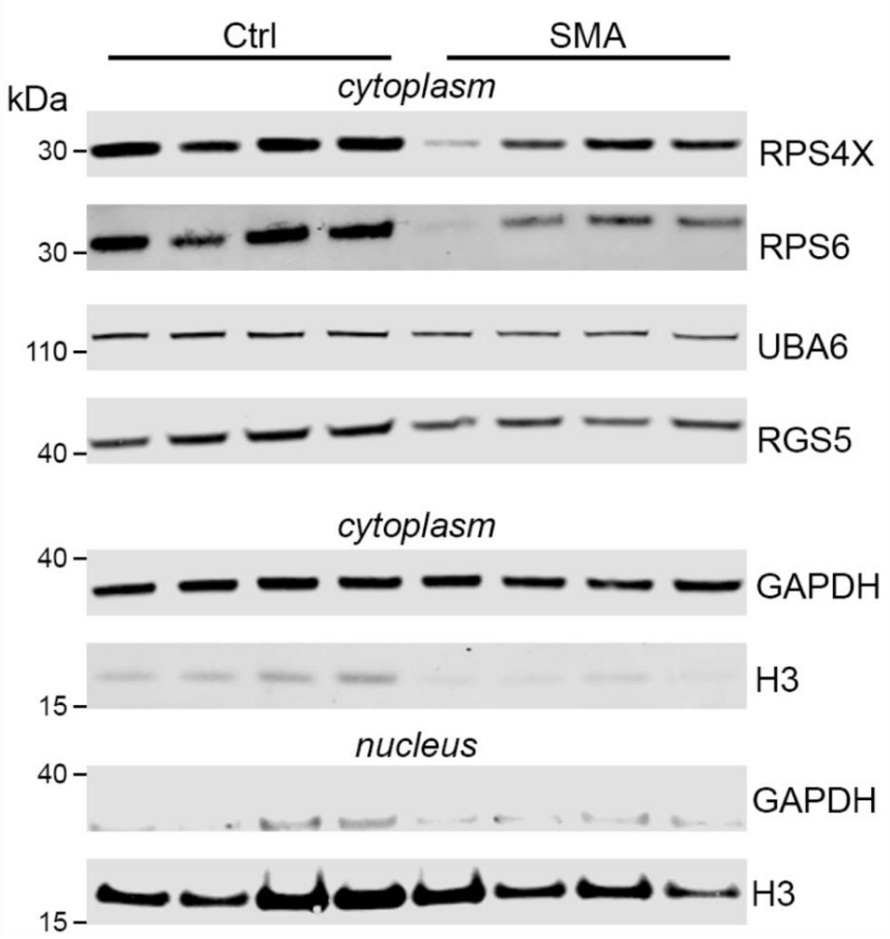

Figure S5. Related to Figure 4 and Figure 5.
Validation of translational changes at protein expression levels.
Brains from late symptomatic SMA mice (SMA) and littermate controls (Ctrl) were biochemically fractionated in a (soluble) cytoplasmic and total nuclear fraction. The bottom half shows enrichment for cytoplasmic proteins (top) and nuclear proteins (bottom) as illustrated by cytoplasmic (GAPDH) and nuclear (Histone H3) marker proteins. Two proteins of our initial qPCR validation set (RGS5, UBA6) and two ribosomal proteins (RPS4X, RPS6) were selected for validation and immunoblotted using the indicated antibodies. Molecular weights are indicated on the left side of the blot (kDa).

Supplemental Experimental Procedures

Subcellular fractionation

The subcellular fractionation protocol was modified from Francisco-Velilla et al. (2016). NSC34 cells lysates were prepared from two 10 cm culture dish with 300 µl polysomal lysis buffer (10 mM Tris–HCl at pH 7.5, 10 mM NaCl, 10 mM MgCl2, 1% Triton-X100, 1% Na-deoxycholate, 600 U/ml RiboLock Rnase Inhibitor (Thermo Scientific), 1 mM DTT, 0.2 mg/ml cycloheximide, 5 U/ml Dnase I (Thermo Scientific). Two consecutive centrifuges of 10 minutes at 14.000 RPM at 4°C were performed to remove efficiently cellular debris, nuclei and mitochondria and supernatant was collected (S30 fraction). S30 was then centrifuged at 95.000 RPM (TLA100.2 rotor), obtaining the S100 supernatant and the R pellet (ribosomes plus associated factors). The R pellet was resuspended in 200 µl of high-salt resuspension buffer (5 mM Tris-HCl pH 7.4, 500 mM KCl, 5 mM MgCl2, 2 mM DTT, 290 mM sucrose), loaded in a 40-20% discontinues sucrose gradient and centrifuged at 4°C 95 000 rpm for 2 h using a TLA100.2 rotor. The obtained pellet (fraction of pure ribosomes, RSW) was resuspended in 100 µl of Electrophoresis Sample Buffer (Santa Cruz Biotechnology) and the supernatant, containing the dissociated factors, collected (FSW). The proteins of the S30, S100 and FSW fractions were extracted by methanol/chloroform protocol (Wessel and Flügge, 1984). S30, S100, R, FSW and RSW were finally separated in a 12% SDS-polyacrylamide gel and analyse by Western Blot.

Western Blotting

To quantify the co-sedimentation profiles of SMN along the sucrose gradient, we analysed Western Blots obtained using a semi-quantitative approach based on densitometric measurements (ImageJ). We calculated the percentage of the protein intensity of each fraction along the sucrose gradient as follows: %Pn = 100 * Dpn / sum Dpni, where Pn is the percentage of the protein in the fraction n; Dpn is the density of the protein in the fraction n and N is the total number of fractions. Western blots for NGS validation experiments were analysed using ImageStudio (LICOR).

Biochemical fractionation of tissues

For biochemical fractionation of late-symptomatic mouse brains, a modified version of the protocol by Cox and Emili was used (Cox & Emili, Nat Protocols 2006). Tissue was initially homogenised in a cytoplasmic lysis buffer (0.25 M glucose, 50 mM Tris-HCl, 5 mM $MgCl_2$, 1 mM DTT, spermine, spermidine (both at 25 µg / mL buffer) and protease inhibitors) using a glass dounce homogenizer, and nuclei where pelleted from the soluble cytoplasmic lysate which was subsequently cleared of contaminants by centrifugation. The nuclear pellet was resuspended in the same buffer with 2M glucose, and pure nuclei were isolated by ultracentrifugation on a glucose cushion. Soluble nuclear proteins were extracted in buffer containing 20% glycerol, 0.02M HEPES, 50 mM Tris-HCl, 5 mM $MgCl_2$, 1 mM DTT and protease inhibitors. DNA and the insoluble nuclear protein fraction were pelleted by centrifugation and DNA bound proteins were extracted using the same buffer with 1% triton-X100. Both nuclear lysates were combined and analysed as total nuclear fraction by Western blot.

Supplemental References

Conway, J.R., Lex, A., and Gehlenborg, N. (2017). UpSetR: An R Package for the Visualization of Intersecting Sets and their Properties. Bioinformatics.

Cox B, Emili A. Tissue subcellular fractionation and protein extraction for use in mass-spectrometry-based proteomics. Nat Protoc. 2006;1(4):1872-8.
